# Structure and function of Gab2 and its role in cancer (Review)

**DOI:** 10.3892/mmr.2015.3951

**Published:** 2015-06-17

**Authors:** CHEN-BO DING, WEI-NA YU, JI-HONG FENG, JUN-MIN LUO

**Affiliations:** 1 Department of Immunology and Immunology Innovation Base for Postgraduate Education in Guizhou Province, Zunyi Medical University, Zunyi, Guizhou 563099, P.R. China; 2 Department of Oncology, Affiliated Hospital of Zunyi Medical University, Zunyi, Guizhou 563099, P.R. China

**Keywords:** Gab2, structure, signal transduction, cancer

## Abstract

The docking proteins of the Grb-associated binder (Gab) family transduce cellular signals between receptors and intracellular downstream effectors, and provide a platform for protein-protein interactions. Gab2, a key member of the Gab family of proteins, is involved in the amplification and integration of signal transduction, evoked by a variety of extracellular stimuli, including growth factors, cytokines and antigen receptors. Gab2 protein lacks intrinsic catalytic activity; however, when phosphorylated by protein-tyrosine kinases (PTKs), Gab2 recruits several Src homology-2 (SH2) domain-containing proteins, including the SH2-containing protein tyrosine phosphatase 2 (SHP2), the p85 subunit of phosphoinositide-3 kinase (PI3K), phospholipase C-γ (PLCγ)1, Crk, and GC-GAP. Through these interactions, the Gab2 protein triggers various downstream signal effectors, including SHP2/rat sarcoma viral oncogene/RAF/mitogen-activated protein kinase kinase/extracellular signal-regulated kinase and PI3K/AKT, involved in cell growth, differentiation, migration and apoptosis. It has been previously reported that aberrant Gab2 and/or Gab2 signaling is closely associated with human tumorigenesis, particularly in breast cancer, leukemia and melanoma. The present review aimed to focus on the structure and effector function of Gab2, its role in cancer and its potential for use as an effective therapeutic target.

## 1. Introduction

Gab2 belongs to the Grb-associated binder (Gab) family of docking proteins, which also includes mammalian Gab1, Gab3 and Gab4, *Drosophila* daughter of sevenless, and *Caenorhabditis elegans* suppressor of Clr-1 (1). Gab2 is reported to contain a pleckstrin homology (PH) domain at the N-terminus, several proline-rich motifs (PXXP) and multiple tyrosine residues, which couple with SH2-containing molecules in a phosphorylation-dependent manner (1,2). The Gab2 PH domain preferentially combines phosphatidylinositol 3,4,5-P_3_ (PIP3) (3). In Gab2, two of the proline-rich motifs are Grb2-Src homology (SH3) domain binding sites (4), which are vital for binding Gab2 to upstream receptors through the Shc-Grb2 complex (5). Gab2 was initially identified as a major binding site of SHP2 phosphatase, which is one of SH2 domain-containing tyrosine phosphatases, in interleukin (IL)-3-stimulated hematopoietic cells (6). Subsequently, this adapter protein was found to be widely involved in a variety of other signaling processes, including the erythropoietin, thrombopoietin, stem cell factor receptor (SCFR), Flt-3 ligand, and the T-cell and B-cell antigen receptor (TCR and BCR, respectively) signaling pathways (7–9).

Gab2 is not only important in signaling systems, but it is important in other physiological activities. Overexprssion of Gab2 enhances the activation of cytokine-dependent extracellular signal-regulated kinase (ERK) mitogen-activated protein kinase (MAPK) and gene expression (9,10). Gab2−/− mice are viable and generally healthy; however, the response of Gab2-knockout mast cells to stimulation via the high affinity lgE receptor (FcεRI) is defective (11). It has been demonstrated that Gab2 adaptor function is intrinsically required for the response of hematopoietic cells to early-acting cytokines, resulting in defective hematopoiesis in Gab2-deficient mice (12). In addition to a role in abnormal development, Gab2 is increasingly being described as associated with mammary tumorigenesis and hematological malignancies. Gab2 is essential for epidermal growth factor (EGF) signaling and breast cancer cell proliferation (13,14). Gab2 has also been considered as a key intracellular intermediate for leukemic transformation, mediated by BCR-ABL (15), and Gab2 is pivotal in the expansion of Friend virus-infected erythroid progenitor cells (16). In the present review, the role of Gab2 protein in signal transduction and its emerging role in cancer are discussed.

## 2. Structure, recruitment and function of Gab2

**Figure 1:**
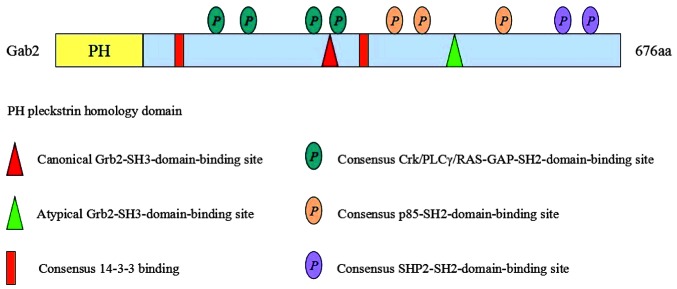
Structure of Gab2 scaffolding protein. The N-terminal PH domain is the most conserved, and its binding to phosphatidyl-inositol-phosphates can promote the membrane recruitment and subcellular localization of Gab2. The central praline-rich domain contains numerous PXXP motifs, which are the binding sites for SH3 domain-containing molecules. C-terminal multiple tyrosines are the binding sites for SH2-domain-containing proteins, including SHP2 and p85. Gab2, Grb-associated binder-2; SHP2, SH2-containing protein tyrosine phosphatase 2; PLC γ, phospholipase C-γ; RAS, rat sarcoma viral oncogene; GAP, GTPase activating protein; SH2 Src homology 2.

The Gab2 gene is located on chromosome 11q13.4-q13.5 in humans, and the molecular weight of Gab2 protein is 97–100 kD. Gab2 is expressed ubiquitously at high levels, particularly in the brain, kidney, lung, heart, testis and ovary (2). Gab2 contains an N-terminal Pleckstrin homology (PH) domain, a central praline-rich domain (PRD) and multiple tyrosines within potential binding motifs, which are favored by various SH-2 and 3 domain-containing proteins (Fig. 1) (2,17). All three domains, particularly the PH domain, are highly conserved in the process of organic evolution.

The N-terminal PH domain is the most conserved, and its binding to PIP3 is involved in membrane recruitment of Gab2. Previous reports have indicated that PH domain may also be involved in regulating intracellular signaling (Fas-signaling pathway), not just a localization module (1,18). The PRD contains numerous PXXP motifs, mediating the interaction with SH3 domain-containing proteins, including Grb2. As shown in Fig. 1, there are multiple sites of tyrosine phosphorylation, which may interact with SH2 domain-containing proteins, including SHP2 and p85. This interaction is important for the function of Gab2 in mediating intracellular signaling pathways, which are crucial for normal cell growth, differentiation, development and apoptosis (19).

## 3. Gab2 in signal transduction

Gab proteins integrate and amplify signals from cytokines, growth factors and antigen receptors, as well as from cell adhesion molecules. They also diversify signals by channeling the input information from activated receptors into signal pathways with distinct biological functions (1). The interactions of Gab2 with other signaling molecules are dependent on its phosphorylation status. In unstimulated cells, Gab2 is located in the cytoplasm, while upon activation by growth hormone (GH), EGF, IL-2/3/15, granuloctye-stimulating factor, interferon (IFN), or T/B cell receptors (20,21), Gab2 can be recruited to the cell membrane by combining with PIP3 via the PH domain. Subsequently, the PRD of Gab2 can interact with Grb2 to from a Gab2-Grb2-Shc complex, which mediates rapid tyrosine phosphorylation. The activated Gab2 contains several docking sites for certain key SH2 domain-containing molecules, including the tyrosine phosphatase, SHP2, and the p85 of subunit of phosphatidylinositol 3-kinase (PI3K), the recruitment and activation of which are induced. At present, the SHP2/rat sarcoma viral oncogene (RAS) and PI3K/AKT pathways are considered to be the two major effector arms of the Gab2 protein.

In the Gab2-SHP2 mediated RAS/ERK pathway, the tyrosine phosphatase, SHP2, is an important binding effector of Gab2 downstream, which contains tandem SH2 domains, the most N-terminal of which confers auto-inhibition of the C-terminal phosphatase domain (22). Gab2 protein contains two SHP2 binding sites, which, if phosphorylated, act as a bisphosphoryl tyrosine activation motif (BTAM) and confers simultaneous binding of the two SH2 domains, thereby relieving auto-inhibition and activating the RAS/ERK signaling pathway (22,23). Thus, SHP2 interaction partners, including Gab2 protein may act, not only as a recruitment platform, but also as an allosteric activator. Gab2 tyrosine phosphorylation site coupling with SH2 domains to activate SHP2 and Gab2 protein regulates diverse biological endpoints, including cell adhesion and the migration of Ba/F3 haematopoietic cells (6), epithelial morphogenesis in MDCK cells (24) and acinar growth of MCF-10A mammary epithelial cells (14). In addition, in certain cellular contexts, the Gab2-SHP2 complex positively regulates other downstream pathways, including c-Kit-induced RAC activation (25) and β1-integrin- and growth factor-induced PI3K activation (6,14).

For the PI3K/AKT signaling pathway, Gab2 has three important tyrosine residues, Y452, Y476 and Y584 sites, for the p85 regulatory subunit of PI3K, which induces the activation of PI3K (18). Activated PI3K leads to the production of phosphatidylinositol-phosphates (PIPs), which bind to the PH domain of Gab2, enhancing the recruitment of Gab2 and promoting the activation of PI3K (26). Thus, a positive feed-back loop is formed to amplify the PI3K/AKT signaling pathway, and the mechanism to produce specific physiological effects is important. It has been demonstrated that Gab1/Gab2 regulates cell survival via the SHP2/ERK and PI3K/AKT pathways in B-cells, and a low level of PI3K activity inhibits Gab2-SHP2 interaction (18), suggesting that PI3K activity is essential for the Gab2/SHP2/ERK signaling pathway.

**Figure 2:**
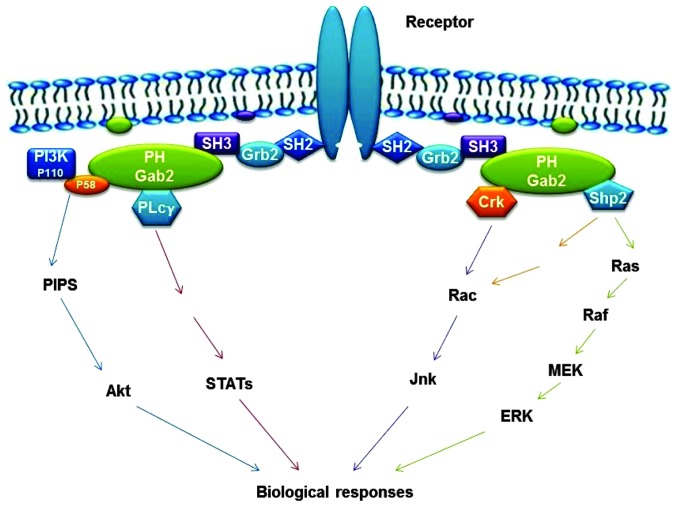
Schematic diagram of the roles of Gab2 protein in signal transduction (1). Characteristics of the mechanism are that the phosphotyrosine residues within the cytoplasmic tails of the activated surface receptors act as binding sites for the SH2-domain of Grb2, which then connects Gab2 via its C-terminal SH3-domain. Activated receptors lead to tyrosine phosphorylation of Gab2 protein and subsequent recruitment of SH2-domain-containing effectors, including SHP2, P85, PLCγ, Crk and STATs. PH-domain confers recruitment of Gab2 to plasma membrane patches enriched in phosphatidyl-inositol-phosphates. Gab2, Grb-associated binder-2; SH2 Src homology 2; SHP, SH2-containing protein tyrosine phosphatase; PLC γ, phospholipase C-γ; RAS, rat sarcoma viral oncogene; Erk, extracellular signal-regulated kinase; MEK, mitogen-activated protein kinase kinase; Jnk, Janus kinas; STAT, signal transducer and activator of transcription; PI3K, phosphatidylinositol 3-kinase.

In addition to the binding sites for SHP2 and p85, Gab2 also contains numerous YXXP motifs, the potential binding sites for Crk family proteins, which are responsible for c-Jun N-terminal kinase (JNK) activation (27). Yu M *et al* demonstrated that Gab2, via its association with SHP2, is required for SCF-evoked activation of the RAC/JNK pathway and mast cell proliferation (25). Biochemical analyses and genetic investigations, as well as yeast-two-hybrid (Y2H) screens have also identified additional Gab effector proteins (Fig. 2), including PLcγ (28), Crk families (29,30), adaptor proteins of the Shc (10), SHIP lipid phosphatase (31), Ras-GTPase activating protein (RasGAP) (32), GC-GAP (31) and the transcriptional activators, signal transducer and activator of transcription (STAT)3 and STAT5 (33,34). However, the detailed mechanism of these effectors interact with Gab2 remain to be fully elucidated.

## 4. Gab2 in cancer

### Breast cancer

It has been reported that the expression of Gab2 is reduced in invasive cancer and lymph node metastases, compared with ductal carcinoma *in situ* (DCIS), although it remains higher than in normal breast tissue (35). Overexpression of Gab2 in MCF-10A cells, an immortalized, non-transformed human mammary epithelial cell line, contributes to increased proliferation and alterations in dependency on EGF and other growth factors (14). By contrast, ablation of Gab2 in several breast cancer cell lines, inhibiting genomic amplifications, leads to a decrease in proliferation, due to a reduction in cell-cycle progression and increased apoptosis, and a reduction in their invasive potential (36). Although the mechanisms by which Gab2 contribute to breast cancer remain to be fully elucidated, the recruitment of SHP2 and subsequent activation of the RAS/MAPK pathway are reported to be required (13). Overexpression of Gab2 in MCF-10A cells promotes enhanced cell migration by modulating the activation of Rashomolog gene family, member A, which is dependent on the SHP2-binding sites (37). Previous investigation has suggested that the RAS/MAPK pathway modulates SHP2 recruitment in a p90 ribosomal S6 kinase (RSK)-dependent manner, and RSK-mediated Gab2 phosphorylation inhibits mammary epithelial cell migration (38).

Gab2 is required for efficient ErbB2-driven mammary tumorigenesis and metastatic spread (13,39). Gab2 acts downstream of Neu, also termed ErbB2 and HER2, and is tyrosyl-phosphorylated upon activation of signal transduction (13). Gab2 and ErbB2 are co-amplified in a subset of breast carcinoma, and co-expression of Gab2 with ErbB2 results in an invasive phenotype, and increases proliferation of MCF-10A mammary cells in a three-dimensional culture. This effect is mediated through downstream SHP2/ERK signaling and is independent of PI3K/AKT activation (13). Agents that interact with the Gab2 or Gab2-mediated pathways may be useful for treating breast tumors overexpressing Gab2 and/or HER2. Several studies using the MCF-10A model system and transgenic mouse models have indicated that, in addition to the above-mentioned HER2, Gab2 also cooperates with other oncogenes linked to the development of breast cancer, including the SRC family.

The small interfering (si)RNA-mediated silencing of Gab2 in breast cancer lines exhibiting Gab2 amplification has suggested a dependency on Gab2 for cell proliferation, cell-cycle progression, survival and invasion, which is likely mediated through altered PI3K and MAPK signaling (36). Qian P *et al* also observed that the p44/42 MAPK-matrix metalloproteinase (MMP)-2/MMP-9 pathway can be used to enhance mammary carcinoma cell migration and invasion consequent to let-7 g depletion by increasing the expression of Gab2 and fibronectin1 (40). In addition, the inhibition or knockdown of the expression of JNK2 in mammary cancer cells reduces tumor cell invasion, and JNK2 conveys these effects in response to a variety of receptor tyrosine kinases, expressed by breast cancer cells by regulating the expression of Gab2 and its downstream signaling (41). These findings highlight a novel role for the Gab2 protein and its role in signaling regulation as a primary genetic diver of breast tumorigenesis.

### Melanoma

As mentioned above, Gab2 is a scaffolding protein that mediates interactions with various signaling pathways, including RAS/ERK and PI3K/AKT signaling. The development of melanoma is inextricably associated with oncogenic activation of these signaling pathways (42,43). Metastatic melanomas express significantly higher levels of Gab2, compared with primary melanomas and melanocytic nevi, identifying Gab2 as a molecular marker for neoplastic progression (44). Furthermore, Gab2 promotes tumor cell migration and invasion by activating PI3K/AKT signaling, and enhances tumor growth and metastasis *in vivo*, suggesting a role for Gab2-mediated signaling in promoting metastatic capability in melanoma (45).

Neuroblastoma v-ras oncogene homolog (NRAS) and v-raf murine sarcoma viral oncogene homolog B1 (BRAF) are oncogenes in melanoma, which are critical for tumor initiation (46). Oncogenic mutations in NRAS can activate the MAPK and PI3K/AKT pathways, whereas mutant BRAF activates the MAPK pathway (47). Gab2 amplification is associated with melanoma arising from sun-protected sites and often occurs independently from oncogenic NRAS or BRAF mutations or amplifications of the KIT gene (44). However, Gab2 is co-expressed with NRAS in melanoma cell lines and tumor samples, and its expression correlates with metastatic potential. Overexpression of Gab2 leads to increased metastatic potential with anchorage independence in soft agar, and a previous report revealed that the cooperative activity of Gab2 in NRAS-driven melanoma increases anchorage independent growth by improving survival of Gab2-expressing cells, enhancing tumorigenesis *in vivo* and facilitating an angiogenic switch through the upregulation of HIF-1a and VEGF by MAPK signaling, but not PI3K signaling, in Gab2/NRAS-driven tumorigenesis (47).

### Ovarian cancer

Compared with its role in breast cancer and melanoma, the function of Gab2 in ovarian carcinoma is less well understood. Genomic amplifications of Gab2 have been described in ~16% of ovarian carcinoma cases (48). The expression of Gab2 predominantly regulates the migratory behaviors of ovarian cancer cells, and overexpression of Gab2 enhances migration and invasion, and downregulates the expression of E-cadherin in ovarian cancer cells with low baseline expression levels of Gab2. Conversely, silencing of Gab2 inhibits the migration and invasion, and positively regulates E-cadherin expression in ovarian cancer cells with high-Gab2 expression (49). The neuregulin/ErbB3 signaling module is important for activation of the PI3K pathway, and promotes cell growth in a subset of ovarian cancer (50). In addition, a previous study has reported that the overexpression of Gab2 activates the epithelial-mesenchymal transition program through activation of the PI3K/Zeb1 pathway, and inhibits the expression of E-cadherin in a subset of ovarian cancer (49). Notably, the OVCAR5 cell lines used in this study exhibit no ErbB3 signaling, and the TOV21G and lgrov-1 cells express low levels of ErbB3 protein, suggesting that the expression of erbB3 and Gab2 contribute to activation of the PI3K pathway in different subsets of ovarian cancer (50). Therefore, the Gab2 acted as ErbB3, which is important in ovarian cancer.

Dunn GP *et al* also identified that Gab2 as an ovarian cancer oncogene, which potently transforms immortalized ovarian and fallopian tube secretory epithelial cells through the activation of PI3K signaling (51). The novel Gab2/PI3K/Zeb1 pathway can be targeted by PI3K and mammalian target of rapamycin (mTOR) inhibitors and can be potentially used to treat Gab2-driven ovarian cancer in combination with standard chemotherapy. In addition, a previous clinical study indicated that novel candidate genes, including UR11, Gab2 and PAK4, may be specifically targeted for the treatment of high-grade serous and endometrioid types of ovarian tumor (52).

### Leukemia

The first evidence for the critical contribution of Gab2 to leukemogenesis was an investigation, which demonstrated that myeloid progenitors from Gab2-deficient mice are resistant to transformation by the BCR-ABL oncoprotein, which arises from a chromosomal translocation found in >90% of patients with chronic myeloid leukaemia (CML). The oncogenic protein tyrosine kinase, BCR-ABL, the product of the Philadelphia chromosome, interacts with Grb2 and Gab2 signaling, and triggers hematopoietic cell proliferation (53). In BCR/ABL-positive CML bone marrow, Gab2-positive myeloid cells are significantly more frequent, compared with normal bone marrow (53). These findings indicate that Gab2 is part of a protein complex that is important, if not essential, in BCR/ABL-driven CML. In addition, BCR-ABL1 is not only present in CML patients, but also occurs in 20–30% of patients with acute lymphoblastic leukemia (ALL) (54,55). Gab2 is an important signal transducer of BCR-ABL1, which combines growth factor and cytokine receptors with downstream effectors, including the PI3K/AKT/mTOR, SHP2/RAS/ERK and JAK/STAT pathways (54). Gab2 does not possess any intrinsic catalytic activity; however, by coupling to effector molecules with distinct enzymatic properties, it leads to the amplification, integration and diversification of the BCR/ABL-derived signaling (1,15). Following recruitment, Gab2 becomes tyrosine phosphorylated, binds to effector proteins, includng PI3K and SHP2 and activates the AKT and ERK signaling pathways (1,56,57). In a similar manner, Gab2 is also involved in multiple nonreceptor tyrosine kinase-linked signaling networks, mediated by erythropoietin or granulocyte colony-stimulating factors receptors (9,58). The pivotal role of Gab2 in BCR-ABL signaling is further demonstrated by observations that short hairpin (sh) RNA-mediated silencing of endogenous Gab2 inhibits the proliferation and colony formation of CD34^+^ cells from patients with CML, but not in cells isolated from healthy donors (59). These findings suggest that human CML may depend on BCR/ABL-driven Gab2 signaling and identify Gab2 as a potential therapeutic target. In addition, a study by Zatkova A *et al* indicated that, in addition to the mixed lineage leukemia gene, Gab2 is a novel candidate target gene of chromosome arm 11q amplification in acute myeloid leukemia (AML)/myelodysplastic syndrome (60).

Despite the significant clinical success of BCR-ABL tyrosine kinase inhibitors (TKIs) in the treatment of CML, mechanisms of TKI-resistance have evolved resulting in CML remaining one of the most difficult types of cancer to treat (61). SHP2 and Gab2 have been demonstrated to be required for BCR/ABL-induced myeloid transformation and leukemia cell proliferation, suggesting that the Gab2-SHP2 axis is an important signaling event in leukemia (15,59). Enhanced sensitivity to the inhibition of Gab2, SHP2 and STAT5 has been observed in BCR/ABL-transformed cell lines. Phosphorylated Gab2 Y452, a PI3K recruitment site, confers Gab2-mediated TKI resistance, while Gab2 knockdown or haploinsufficiency increases TKI sensitivity (55). Ding J *et al* also indicated that SUP-B15, a Ph+ cell line, expresses unusually high levels of Gab2, potentially causing TKI resistance (54). Constitutive phosphorylation of SHP2 is associated with the binding of SHP2 with the p85 PI3K regulatory subunit and Gab2, which is sufficient for KITD814V-induced myeloproliferative disease (MPD). By contrast, the SHP2 inhibitor enhances the efficacy of the PI3K inhibitor in suppressing KITD814V-induced ligand-independent growth *in vitro* and MPD *in vivo* (62). Furthermore, targeting of the N-SH2 domain of SHP2 with monobodies markedly reduces its interaction with Gab2 and has significant effects on downstream signaling in BCR/ABL-drived CML (63). These findings suggest that the Gab2-SHP2 axis may be exploited as a novel modulator of TKI sensitivity in CML and as a potential therapeutic target in TKI-resistant disease. Notably, a previous study demsontrated that, at equimolar concentrations, dasatinib is more effective in preventing Gab2 tyrosine and serine/threonine phosphorylation, compared with imatinib, suggesting that dasatinib may be an alterative in the clinical therapy of CML (64).

BCR-ABL stability and oncogenic signaling in CML cells are under the control of Janus kinase-2 (JAK2) (65). The inhibition of JAK2 reduces the levels of tyrosine phosphorylation of Shc and Gab2, and reduces activation of the RAS, PI3K and STAT5 pathways, thereby inducing apoptosis in CD34^+^ cell from patients with CML in blast crisis (65). STAT5 is a critical transcription factor for normal hematopoiesis, and its sustained activation is connected with hematological malignancy. A persistently active mutant of STAT5 (STAT5a^s711F^) associates with Gab2 in myeloid leukemias and promotes growth *in vitro* via the activation of AKT activation (66). Nagao T *et al* found that apoptosis may be suppressed in PVTL-1 cells, an AML cell line, through inactivation of GSK3 by Lyn, and of JAK2-V617F and is also suppressed by the activation of STAT5 by JAK2-V617F (67). Tyrosine-phosphorylated-STAT5 can be tracked using flow cytometry or immunostaining and is a biomarker associated with poor prognosis in patients with juvenile myelomonocytic leukemia (JMML) (66) and AML (68).

JMML, an MPD of young children characterized by cytokine hypersensitivity of myeloid progenitors, is associated with a mutation in the RAS pathway (69,70). PTPN11 is the most common target of genetic mutations in JMML (71,72), and 35% of patients with JMML have activating mutations in tyrosine phosphatase PTPN11 (SHP2), a known positive regulation of the RAS pathway. These mutations, including the congenital mutation D61 G and somatic mutation E76K, disrupt the inhibitory intramolecular interaction between the N-terminal SH2 (N-SH2) and catalytic domains, leading to hyperactivation of SHP2 (71,73). Furthermore, interactions of mutant SHP2 with tyrosine-phosphorylated signaling partners, including Gab1 and Gab2, are enhanced by mutations in the N-SH2 domain (74,75). Gab2 as an important regulatory protein and is vital in the mutant SHP2-mediated RAS pathway. In addition, SHP2 mutations and the SHP2 binding protein, Gab2, are associated with hyperactivation of the ERK, AKT and STAT5 pathways in JMML, suggesting novel approaches to JMML therapy (76).

### Gab2 in other types of malignancy

Gab2 is overexpressed in malignant lung tissues, compared with normal lung tissues, suggesting Gab2 has a novel role in the development of lung cancer (77). The association between c-Met, and PI3K and Gab2 in small cell lung cancer enhances cell motility and invasion as an important consequence of c-Met signaling (78). In addition, a previous study reported that Gab2 positively regulates mucin synthesis and goblet cell hyperplasia through an IL-13-mediated TYK2/STAT6 pathway in lung cancer and chronic obstructive pulmonary disease (79). In addition, Gab2-mediated signaling may result in the activation of AKT and promote invasion in glioma cell via the AKT/mTOR pathway (80). Lee SH *et al* demonstrated that Gab2 is over-expressed in malignant gastric cells, compared with normal epithelial cells, suggesting that the expression of Gab2 may be involved in the development of gastric cancer (81). The aforementioned evidence suggests that the overexpression of Gab2 and its signaling are important in human malignancies, however, additional functional investigations are required to identify more key proteins, which combine with Gab2, and are involved in the proliferation, differentiation and migration of tumor cells. This is vital for understanding carcinogenesis and devising novel therapeutic approaches.

## 5. Conclusion and perspective

As summarized in the present review, it has been demonstrated over several years that numerous kinases and phosphorylation events regulate Gab2 signal transduction, the signaling of which is summarized in Fig. 2. The pathophysiology of the expression of Gab2 and/or Gab2 signal transduction is complex and dependent upon oncogenic processes and host cell biological responses. Although a body of evidence has demonstrated that aberrant Gab2 and/or Gab2 signaling is closely associated with malignant biological properties of tumors, particularly in breast and ovarian cancer, melanoma and leukemia, the detailed mechanism remains to be fully elucidated. Therefore, further investigations are warranted to improve understanding.

Notably, the EMT and cancer stem cells (CSCs) are also critical in cancer pathogenesis (82). Activation of the EMT triggers tumor cell invasion and metastasis to distant organs via the downregulation of intercellular adhesion molecules, including E-cadherin and occludin, and the upregulation of mesenchymal markers, including vimentin and N-cadherin (83). The EMT is also involved in the acquisition of CSC properties, and EMT-inducing CSCs have been considered an important origin of CSCs (82). In addition to their capacities in tumor initiation, CSCs have also been implicated in tumor invasion and metastasis (83). Improving understanding of the mechanism underlying the activation of different signaling pathways by Gab2 in promoting tumor cell metastasis, migration and recurrence, combined with current understanding of EMT and CSCs, may provide novel insight for designing effective therapies to treat different types of cancer.
